# Trend and early outcomes in isolated surgical aortic valve replacement in the United Kingdom

**DOI:** 10.3389/fcvm.2022.1077279

**Published:** 2023-01-09

**Authors:** Jeremy Chan, Arnaldo Dimagli, Daniel P. Fudulu, Shubhra Sinha, Pradeep Narayan, Tim Dong, Gianni D. Angelini

**Affiliations:** ^1^Bristol Heart Institute, University of Bristol, Bristol, United Kingdom; ^2^NH Rabindranath Tagore International Institute of Cardiac Sciences, Kolkata, India

**Keywords:** aortic valve, trend, surgical aortic replacement, transcatheter and surgical aortic valve replacement, United Kingdom

## Abstract

**Objective:**

Surgical aortic valve replacement (SAVR) is traditionally the gold-standard treatment in patients with aortic valve disease. The advancement of transcatheter aortic valve replacement (TAVR) provides an alternative treatment to patients with high surgical risks and those who had previous cardiac surgery. We aim to evaluate the trend, early clinical outcomes, and the choice of prosthesis use in isolated SAVR in the United Kingdom.

**Methods:**

All patients (*n* = 79,173) who underwent elective or urgent isolated surgical aortic valve replacement (SAVR) from 1996 to 2018 were extracted from the National Adult Cardiac Surgery Audit database. Patients who underwent additional procedures and emergency or salvage SAVR were excluded from the study. Trend and clinical outcomes were investigated in the whole cohort. Patients who had previous cardiac surgery, high-risk groups (EuroSCORE II >4%), and predicted/observed mortality were evaluated. Furthermore, the use of biological prostheses in five different age groups, that are <50, 50–59, 60–69, 70–79, and >80, was investigated. Clinical outcomes between the use of mechanical and biological aortic valve prostheses in patients <65 years old were analyzed.

**Results:**

The number of isolated SAVR increased across the study period with an average of 4,661 cases performed annually after 2010. The in-hospital/30-day mortality rate decreased from 5.28% (1996) to 1.06% (2018), despite an increasing trend in EuroSCORE II. The number of isolated SAVR performed in octogenarians increased from 596 to 2007 (the first year when TAVR was introduced in the UK) to 872 in 2015 and then progressively decreased to 681 in 2018. Biological prosthesis usage increased across all age groups, particularly in the 60–69 group, from 24.59% (1996) to 81.87% (2018). There were no differences in short-term outcomes in patients <65 years old who received biological or mechanical prostheses.

**Conclusion:**

Surgical aortic valve replacement remains an effective treatment for patients with isolated aortic valve disease with a low in-hospital/30-day mortality rate. The number of patients with high-risk and octogenarians who underwent isolated SAVR and those requiring redo surgery has reduced since 2016, likely due to the advancement in TAVR. The use of biological aortic prostheses has increased significantly in recent years in all age groups.

## Introduction

Surgical aortic valve replacement (SAVR) has been the treatment for patients with aortic valve disease since it was first performed in 1960 (1, 2), with biological and mechanical prostheses most commonly used to replace the native diseased valve. The long-term outcome and freedom from structural valve degeneration for SAVR remain excellent (3, 4). However, the choice of the ideal valve in relation to the specific age group remains controversial.

The recent advancement of transcatheter aortic valve replacement (TAVR) provides an alternative treatment. The PARTNER 1, 2, and 3 trials have shown the benefits of TAVR in all patients with aortic stenosis, regardless of surgical risk (5–7). TAVR also provides an alternative to patients with the previous SAVR/TAVR with an option of valve-in-valve transcatheter aortic valve replacement (ViV TAVR). This has been suggested to result in a reduction in mechanical valve use worldwide (8, 9).

We aim to evaluate the trend and clinical outcomes in isolated SAVR in the United Kingdom and also focus on the volume of SAVR in the high-risk cohort, redo surgery, and the choice of prosthesis use. Finally, early clinical outcomes in patients who received mechanical or biological prostheses under the age of 65 were compared.

## Materials and methods

All patients undergoing elective or urgent isolated SAVR in the United Kingdom from 1996 to 2018 were extracted from the National Adult Cardiac Surgery Audit (NACSA) database. The NACSA database prospectively collects data on all major heart operations conducted on National Health Service patients in the United Kingdom since April 1996. The definitions of database variables were used, and a description of the database was previously described (10).

Patients were divided into five different age groups: less than 50 years old, 50–59 years old, 60–69 years old, 70–79 years old, and above 80 years old. The surgical volume and choice of aortic valve prostheses (biological vs. mechanical) across the five age groups were investigated. The choice of aortic valve prostheses used in patients with chronic kidney disease undergoing dialysis was also evaluated (As defined in one of the three categories—1. dialysis for acute renal failure: onset within 6 weeks of cardiac surgery, 2. dialysis for chronic renal failure: onset more than 6 weeks prior to cardiac surgery, and 3. no dialysis but pre-operative acute renal failure (anuria or oliguria <10 ml/h). The trend in patients who underwent previous cardiac surgery and the volume of SAVR performed in patients with low- (<2%), intermediate- (2–4%), and high-risk (>4%) as defined by the EuroSCORE II was also evaluated (11). The observed mortality was defined as 30-day or in-hospital mortality after the index operation. The expected mortality was calculated using the EuroSCORE II. Moreover, the early clinical outcomes in patients who received mechanical or biological prostheses under the age of 65 were compared using propensity score matching. Sixty-five was selected as a cutoff based on the 2020 ACC/AHA Guideline for the Management of Patients with Valvular Heart Disease (12).

Patients who underwent additional procedures, major aortic surgery, emergency, or salvage SAVR were excluded from the study. Missing data in the choice of prostheses were also excluded in this study (*n* = 3,309, 4.18%).

### Ethics statement

The study is part of a research project approved by the Health Research Authority (HRA) and Health and Care Research Wales (HCRW). As the study included retrospective interrogation of the NACSA database, the need for individual patient consent was waived off (HCRW) (IRAS ID: 278171) in accordance with the research guidance. The study was performed in accordance with the ethical standards as laid down in the 1964 Declaration of Helsinki and its later amendments. The General Data Protection Regulations were strictly followed for the use of all data.

## Statistical analysis

Continuous variables are reported as mean and standard deviation (SD). Categorical variables are reported as frequencies and percentages. Pearson’s chi-squared test, Wilcoxon rank-sum test, and one-way/multi-factor analysis of variance were used to compare two categorical variables, for comparison between two means of continuous, independent samples, and to compare between three continuous variables, respectively.

Propensity score matching (PSM) was performed to create a quasi-experimental design by balancing measured confounding factors between the two groups. A 1:1 nearest neighbor matching without replacement with a caliper width of 0.2 standard deviations of the logit of the propensity scores was performed using the pre-operative characteristics listed in Table 3. Missing continuous variables data (left ventricular ejection fraction and body mass index) were imputed with the median value in the data after the application the of exclusion criteria listed above. After matching, all standardized mean differences for the covariates were checked. The adequate balance was set to be below 0.1.

The effectiveness of the PSM was visualized with a Love plot to report the covariate balance with all variables before and after the matching. This is shown in the Supplementary Figure 1. Binary logistic regression was performed using the baseline patient demographics and comorbidities to predict factors associated with the use of biological aortic valve prostheses. The results are demonstrated as odds ratio (OR) and 95% confidence interval (95% CI). A *P*-value of < 0.05 is deemed statistically significant.

R (Version 4.1.1) and R Studio (Version 1.4.1103, RStudio, PBC) were used to perform the statistical analysis. The following packages were used: tidyverse, MatchIt, sjPlot, and ggplot2. Graphs and tables were created using R Studio (Version 1.4.1103, RStudio, PBC) and Microsoft Office 365 (Version 16.0.14026, 64 bits).

## Results

A total of 79,173 patients underwent isolated SAVR in the study period. The mean age was 67.99 (SD 12.48) years old, and 32,799 (41%) were female. The mean BMI was 28.35 (SD 5.64), and LVEF was 53.60 (SD 10.89). About 81% (*n* = 64,457) were performed in an elective setting. The median post-operative length of stay was 6 days (IQR 5–7 days). The most common surgical incision was median sternotomy (88.7%), followed by partial/hemisternotomy (9.05%). In patients who underwent minimally access SAVR, 4.9% required conversion to median sternotomy.

## Etiology

The most common etiology was aortic stenosis (66.8%) followed by mixed aortic stenosis and regurgitation (18.3%) and aortic regurgitation (14.9%). The native valve pathology was most likely due to degeneration (76.1%) followed by congenital (9.4%). Infective endocarditis and rheumatic heart disease accounted for 3.6 and 2.3% of total cases, respectively.

### Predicted/observed mortality

**FIGURE 1:**
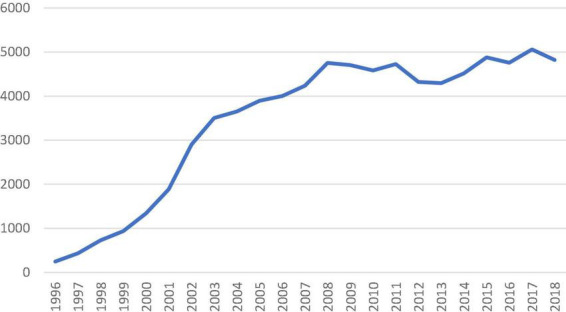
Number of isolated surgical aortic valve replacement performed in the United Kingdom from 1996 to 2018 recorded in the national database.

**FIGURE 2:**
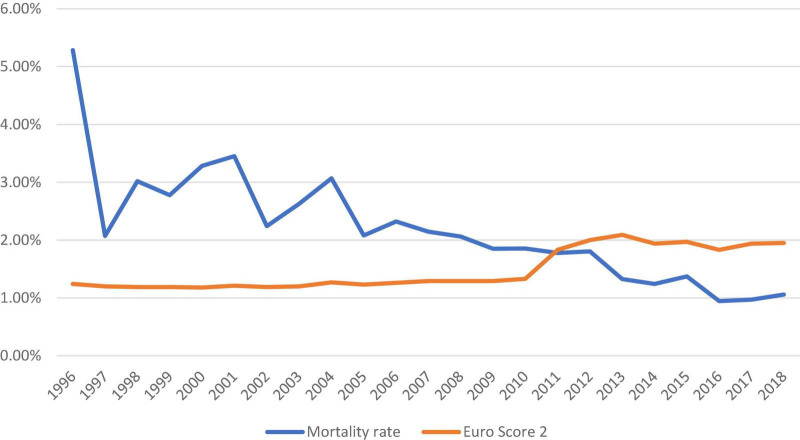
Trend of observed and expected mortality rates in patients who underwent isolated surgical aortic valve replacement in the United Kingdom from 1996 to 2018.

The number of isolated SAVR increased across the study period with an average of 4,661 cases performed annually after 2010 (Figure 1). The in-hospital/30-day mortality rate decreased from 3.28% (2000) to 1.06% (2018), despite an increasing trend in EuroSCORE II. The observed/expected mortality has significantly reduced over the study period and was below 1.0 since 2011. The trend of observed and expected mortality rates is shown in Figure 2.

### Isolated SAVR in octogenarians, intermediate- and high-risk groups, and patients who underwent previous cardiac surgery

The number of isolated SAVR in octogenarians increased from 596 to 2007 (the first year when TAVR was introduced in the UK) to 872 in 2015. Since then, the number of isolated SAVR has reduced to 823, 752, and 681 in 2016, 2017, and 2018, respectively.

**FIGURE 3:**
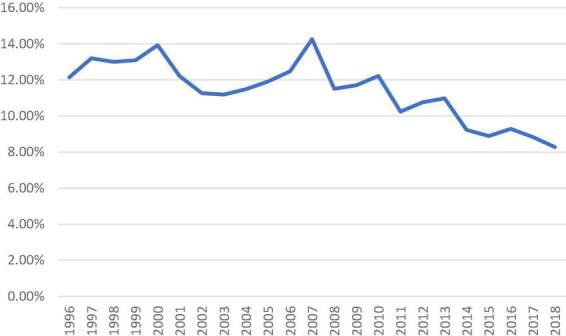
Proportion of isolated surgical aortic valve replacement patients who underwent previous cardiac surgery from 1996 to 2018.

There was an increasing trend of patients with intermediate-risk who underwent isolated SAVR, particularly after 2010 (>20% of all isolated SAVR). In patients with high-risk, a downward trend was observed since 2016. Redo-isolated SAVR peaked in 2007 (14.26% of all isolated SAVR performed) and gradually declined to 8.27% in 2018 (Figure 3).

### Choice of aortic valve prostheses

**FIGURE 4:**
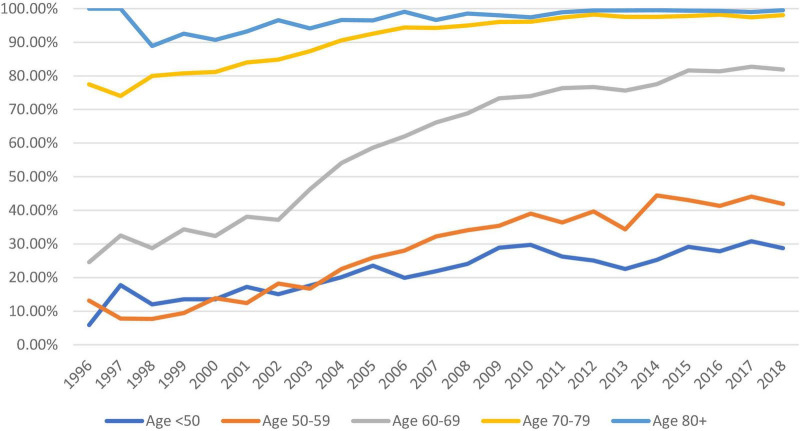
Trend of the proportion of bioprostheses used (against mechanical prostheses) across all five age groups from 1996 to 2018 (dark blue: <50, orange: 50–59, gray: 60–69, yellow: 70–79, and pale blue: >80).

Biological aortic prosthesis use increased across all age groups from 1996 to 2018. Nearly, all patients above 70 s received a biological prosthesis since 2010 (98.09 and 99.52% in the 70–79 and >80 groups, respectively in 2018). The increase in the adoption of biological prostheses was most apparent in the 60–69 groups, from 24.59% to 1996 to 81.87% in 2018. There was also increased use of bioprostheses in patients below the age of 50 (28% in 2018) (Figure 4).

### Choice of aortic valve prostheses in patients with chronic kidney disease requiring dialysis

Eighty-two patients had acute renal failure requiring dialysis within 6 weeks before SAVR; of these, 74.39% (*n* = 61) received a mechanical prosthesis. In patients with chronic renal failure requiring long-term dialysis, 60.2% (*n* = 150) received a mechanical prosthesis. One hundred and four patients (85.95%) with acute renal failure who did not undergo dialysis prior to surgery received a mechanical prosthesis.

### Factors predicting the use of biological aortic valve prostheses

Pre-operative characteristics including age, EuroSCORE II, and patients with pre-operative neurological dysfunction were more likely to receive a biological aortic valve prosthesis. On the other hand, female sex, patients with pre-operative atrial fibrillation and peripheral vascular disease were more likely to receive a mechanical prosthesis (Table 1).

**TABLE 1:** Factors predicting the use of biological (age, LVEF, current smoker, EuroSCORE II, and neurological dysfunction) or mechanical (female sex, urgent operation, peripheral vascular disease, and pre-operative atrial fibrillation) prostheses (CI, confidence interval, BMI, body mass index, LVEF, left ventricular ejection fraction, PVD, peripheral vascular disease, AF, atrial fibrillation).

Pre-operative characteristics	Odd ratios (95% CI)	*P*
Age (year)	1.14 (1.14–1.14)	<0.001
Female sex	0.96 (0.91–1.00)	0.043
BMI	1.00 (0.99–1.00)	0.137
LVEF	1.02 (1.02–1.02)	<0.001
Urgent operation	0.83 (0.78–0.88)	<0.001
Smoking (Current)	1.18 (1.09–1.27)	<0.001
Pulmonary disease	0.94 (0.89–1.00)	0.061
PVD	0.72 (0.65–0.79)	<0.001
Pre-operative AF	0.56 (0.53–0.60)	<0.001
Euro Score II	1.51 (1.46–1.57)	<0.001
Neurological dysfunction	1.57 (1.35–1.82)	<0.001

### Early clinical outcomes between biological and mechanical prosthesis in patients <65 years old

After propensity score matching, there were no differences between patients <65 years old who received either biological or mechanical aortic valve prostheses in mortality, return to the theater (including for bleeding and tamponade), post-operative neurological events, dialysis, and deep sternal wound infection. The aortic cross-clamp time and cardiopulmonary bypass time were lower in patients who received mechanical aortic valves (Table 2). The pre-operative characteristics are shown in Table 3.

**TABLE 2:** Intra- and post-operative outcomes of patients <65 years old receiving biological or mechanical prostheses.

Characteristics	Biological (*n* = 9,246)	Mechanical (*n* = 9,246)	*p*-value
CPB (Minute)	99.17 (45.76)	95.71 (44.04)	<0.001
XClamp (Minute)	74.16 (32.10)	72.11 (29.84)	<0.001
Mortality	81 (0.9%)	86 (0.9%)	0.73
Return to theater	422 (5.2%)	428 (5.1%)	0.93
Postop stroke			0.92
TIA	34 (0.4%)	33 (0.4%)	
CVA	38 (0.5%)	42 (0.5%)	
Postop dialysis	107 (1.3%)	119 (1.4%)	0.62
Postop DSWI	12 (0.5%)	15 (0.5%)	0.85

Data are expressed as mean ± SD. N/A, not applicable, OR, odds ratio, CI, confidence interval, XClamp, cross-clamp, CPB, cardiopulmonary bypass, DSWI, deep sternal wound infection, TIA, transient ischemic attack, CVA, cerebral vascular accident, Postop, post-operative.

**TABLE 3:** Pre-operative characteristics of the biological and mechanical prostheses in patients <65 years old before and after propensity score matching.

Pre-operative characteristics	Pre PSM			Post PSM			
	Biological (*n* = 15,989)	Mechanical (*n* = 9,927)	*P*-value	Biological (*n* = 9,246)	Mechanical (*n* = 9,246)	SMD	*p*-value
Age (Year)	52.53 (9.69)	56.94 (9.01)	<0.001	56.07 (8.54)	56.56 (9.15)	-0.0546	<0.001
Gender (Male)	11,417 (71%)	6,774 (68%)	<0.001	2,890 (31%)	2,875 (31%)	0.0035	0.81
BMI	28.70 (5.85)	28.79 (5.19)	0.14	28.63 (5.64)	28.85 (5.93)	0.0368	0.027
CCS grade			<0.001				0.41
0	9,667 (60%)	6,143 (62%)		5,788 (63%)	5,662 (61%)	0.0281	
1	24, 18 (15%)	1,334 (13%)		1,218 (13%)	1,280 (14%)	-0.0197	
2	2,662 (17%)	1,750 (18%)		1,583 (17%)	1,635 (18%)	-0.0148	
3	967 (6.0%)	563 (5.7%)		527 (5.7%)	541 (5.9%)	-0.0065	
4	275 (1.7%)	137 (1.4%)		130 (1.4%)	128 (1.4%)	0.0019	
NYHA status			<0.001				0.59
1	3,604 (23%)	1,805 (18%)		1,790 (19%)	1,743 (19%)	0.0132	
2	6,638 (42%)	4,325 (44%)		4,049 (44%)	4,011 (43%)	0.0083	
3	4,840 (30%)	3,205 (32%)		2,899 (31%)	2,965 (32%)	-0.0153	
4	907 (5.7%)	592 (6.0%)		508 (5.5%)	527 (5.7%)	-0.0087	
Preop AF	850 (5.3%)	404 (4.1%)	<0.001	341 (3.7%)	388 (4.2%)	-0.0257	0.076
Previous MI			0.36				0.95
No	15,384 (96%)	9,516 (96%)		8,881 (96%)	8,884 (96%)	-0.0016	
1	555 (3.5%)	377 (3.8%)		336 (3.6%)	331 (3.6%)	0.0028	
2 Or more	50 (0.3%)	34 (0.3%)		29 (0.3%)	31 (0.3%)	-0.0037	
Previous PCI			<0.001				0.86
No	15,728 (98%)	9,651 (97%)		9,022 (98%)	9,036 (98%)	-0.0092	
24 h before op	18 (0.1%)	13 (0.1%)		10 (0.1%)	11 (0.1%)	-0.0030	
same admission	9 (<0.1%)	420 (0.8%)		6 (<0.1%)	7 (<0.1%)	-0.0033	
Previous admission	234 (1.5%)	252 (2.5%)		208 (2.2%)	192 (2.1%)	0.0010	
LVEF	53.38 (6.09)	53.49 (713)	<0.001	53.52 (6.21)	53.53 (6.92)	0.0021	<0.001
Diabetes			<0.001				0.79
No	14,547 (91%)	8,682 (87%)		8,167 (88%)	8,163 (88%)	0.0013	
Diet control	265 (1.7%)	219 (2.2%)		186 (2.0%)	199 (2.2%)	-0.0096	
Drug control	823 (5.1%)	722 (7.3%)		634 (6.9%)	613 (6.6%)	0.0087	
Insulin	354 (2.2%)	304 (3.1%)		259 (2.8%)	271 (2.9%)	-0.0075	
Smoking			0.004				0.65
Non-smoker	7,605 (48%)	4,516 (45%)		4,279 (46%)	4,221 (46%)	0.0126	
Ex-smoker	6,709 (38%)	3,956 (40%)		3,651 (39%)	3,709 (40%)	-0.0128	
Current smoker	2,305 (14%)	1,455 (15%)		1,316 (14%)	1,316 (14%)	0.00	
Pulmonary disease	1,704 (11%)	1,250 (13%)	<0.001	1,141 (12%)	1,111 (12%)	0.0098	
NeuroDys	271 (1.7%)	340 (3.4%)	<0.001	231 (2.5%)	225 (2.4%)	0.0036	0.78
PVD	677 (4.2%)	453 (4.6%)	0.21	400 (4.3%)	411 (4.4%)	-0.0057	0.69
Creatinine > 200	282 (1.8%)	200 (2.0%)	0.15	156 (1.7%)	172 (1.9%)	-0.0123	0.37
Euro Score II	1.00 (1.00)	1.18 (1.48)	<0.001	1.06 (1.21)	1.09 (1.11)	-0.0194	<0.001

AF, atrial fibrillation, CCS, canadian cardiovascular society, PCI, percutaneous coronary intervention, BMI, body mass index, NYHA, New York Heart Association, LMS, left main stem disease, MI, myocardial infraction, LVEF, left ventricular ejection fraction, NeuroDys, neurological dysfunction, ES2, EuroSCORE II, PSM, propensity score matching, SMD, standardized mean difference, PVD, peripheral vascular disease.

## Discussion

Our study demonstrated that SAVR remains an effective treatment with low mortality for patients with isolated aortic valve disease. The number of patients with high-risk, octogenarians undergoing isolated SAVR, and those requiring redo surgery has reduced in recent years, likely due to the advancement in TAVR. The use of biological aortic prostheses has increased significantly in recent years in all age groups.

Despite the increment in EuroSCORE II, the overall mortality rate has continued a downward trend to 1.06% in 2018. The observed mortality rate has outperformed the predicted mortality rate by nearly 50% in 2016–2018. The mortality rate in the United Kingdom has further reduced since the last report (13) and other reports in the literature (9, 14).

The increased use of bioprostheses we observed reflects a similar trend in the literature. Jiménez-García et al. (9) reported a five-fold use of bioprostheses from 2001 to 2015 using the Spanish National Hospital Discharge Database, although the age groups were not specified. Alkhouli et al. (8) reported the age group-specific use of bio/mechanical prostheses in the United States using the Nationwide Inpatient Sample. They observed a significant reduction in the use of mechanical prostheses among patients aged 50–70 years between 2008 and 2017. This is similar to our finding in which more than 80% of patients aged 60–69 received a biological aortic valve. However, the use of mechanical valves remains higher in the 50–59 age group in the United Kingdom (58% in 2018) compared with 37 and 22% among the 56–60 and 50–55 age groups in the United States (8).

The durability of bioprostheses remains an important topic when used in young patients. The freedom from structural valve deterioration has been reported at 81.7% at 15 years and 52% at 20 years post-SAVR (15); hence, the need to repeat interventions should be taken into consideration. The advancement of TAVR, valve-in-valve transcatheter aortic valve replacement (ViV TAVR), has become an alternative treatment in patients with the previous SAVR. In addition to avoidance of anticoagulation, these may be the explanation for the observed reduction in the use of mechanical prostheses. However, a higher rate of prosthesis–patient mismatch, paravalvular leak, and coronary obstruction, in ViV TAVR compared to native valve TAVR has been reported (16, 17).

In our report, we observed a higher proportion of mechanical valves used in patients requiring dialysis. With the high risk of early structural deterioration in biological prostheses, there seems to be a preference for the use of the mechanical valve in this subgroup. This is in line with literature reports. Uzuka et al. (18) reported the risk of early structural deterioration in the biological valve to be approximately 50% at 6 years (compared to 0% in the control arm). Chi et al. (19) also demonstrated the survival benefits of implanting mechanical prostheses in patients with dialysis.

Currently, the European guideline recommends that patients above the age of 75 should consider TAVR after Heart/Valve team evaluation (20), while the American guideline had a cutoff of 65 years old (12). The National Institute for Health and Care Excellence recommends SAVR as a first-line interventions and TAVR for patients who are at high surgical risk or if surgery is unsuitable (21). However, with the results of the UK TAVI trials, the authors predicted that the volume of TAVR will continue to increase and exceed SAVR in the United Kingdom (22). Indeed, in the NACSA 2022 reports, the ratio of TAVR to SAVR rose from 1.2:1 to 2.3:1, which dramatically accelerated during the COVID-19 pandemic (23). The annual volume of TAVR has already exceeded SAVR in the USA and Germany (24, 25).

Our study demonstrated similar short-term outcomes regardless of the valve of choice in non-elderly patients, which echo the findings reported in the literature. Most studies showed the risk of re-intervention is higher in bioprostheses although the risk of major bleeding is lower. However, the long-term survival benefits remain controversial with some studies demonstrating a survival benefit with mechanical valves (26–28) while some did not (29–32). Although the survival benefits could be due to primary valve failure, which could be less of an issue due to the advancement of TAVR, particularly in patients who are at high risk for a redo of SAVR.

## Limitations

There are several limitations of the study. First, the impact of SVAR on the clinical practice may not be fully evaluated, in a patient with extra pathology such as mitral valve/aortic/coronary artery disease requiring concomitant procedures. The choice of prostheses in these patients was not evaluated in this report. In addition, patients with aortic stenosis and coronary artery disease may undergo TAVR and their coronary artery disease managed at a later stage, while in the surgical arm, these patients will undergo both coronary artery bypass graft and SAVR at the same time. The morphology (bicuspid and tricuspid) was not coded in the database and remains an important limitation as patients with bicuspid valves are more likely to be referred for a SAVR. The lack of TAVR registry data to compare the volume of SAVR against TAVR is another major limitation.

The lack of pre- and post-operative precise echocardiographic data limited the sub-analysis for patients who underwent SAVR with different pathologies. In addition, the database did not collect certain post-operative outcomes and follow-up data; hence, information on pacemaker implantation, repeat valve intervention, endocarditis, and structural valve deterioration is not available. Finally, the database requires input from all healthcare professionals, missing data are seen in several non-mandatory items, and these are instead presented as a percentage. Overall, the mandatory items, particularly items essential for EuroSCORE II risk stratification, are well maintained with an acceptable rate (<5%) of missing data since the 2,000s.

Despite the application of PSM, it is possible that residual bias is present in the analysis since the propensity-matched model can account only for measured confounders and not for the unmeasured confounders (e.g., frailty).

## Conclusion

Our data show that isolated SAVR is a safe and effective treatment with very low in-hospital mortality for patients with aortic valve disease. The advancement of transcatheter aortic valve replacement is the likely explanation for the reduction of SAVR in patients with high-risk, octogenarians, and those requiring redo surgery. The option of valve-in-valve transcatheter aortic valve replacement may also be one of the causes for the observed use of biological aortic prostheses which has increased significantly in recent years in all age groups.

## Data availability statement

The datasets presented in this article are not readily available because Ethical approval required. Requests to access the datasets should be directed to NICOR, nicor.auditenquiries@nhs.net.

## Ethics statement

The studies involving human participants were reviewed and approved by Health Research Authority (HRA) and Health and Care Research Wales (HCRW) (IRAS ID: 278171). Written informed consent for participation was not required for this study in accordance with the national legislation and the institutional requirements.

## Author contributions

JC: conceptualization, data curation, formal analysis, methodology, writing—original draft, and writing—reviewing and editing. AD: data curation, formal analysis, methodology, writing—original draft, and writing—reviewing and editing. TD: data curation, formal analysis, methodology, and writing—reviewing and editing. DF, SS, and PN: conceptualization and writing—reviewing and editing. GA: conceptualization, supervision, methodology, and writing—reviewing and editing. All authors contributed to the article and approved the submitted version.
