# Supplementary figures and images for: Trend and early outcomes in isolated surgical aortic valve replacement in the United Kingdom
Source: Front Cardiovasc Med. 2023 Jan 9;9:1077279. doi: 10.3389/fcvm.2022.1077279 (PMC9868612; doi:10.3389/fcvm.2022.1077279)

# Covariate Balance

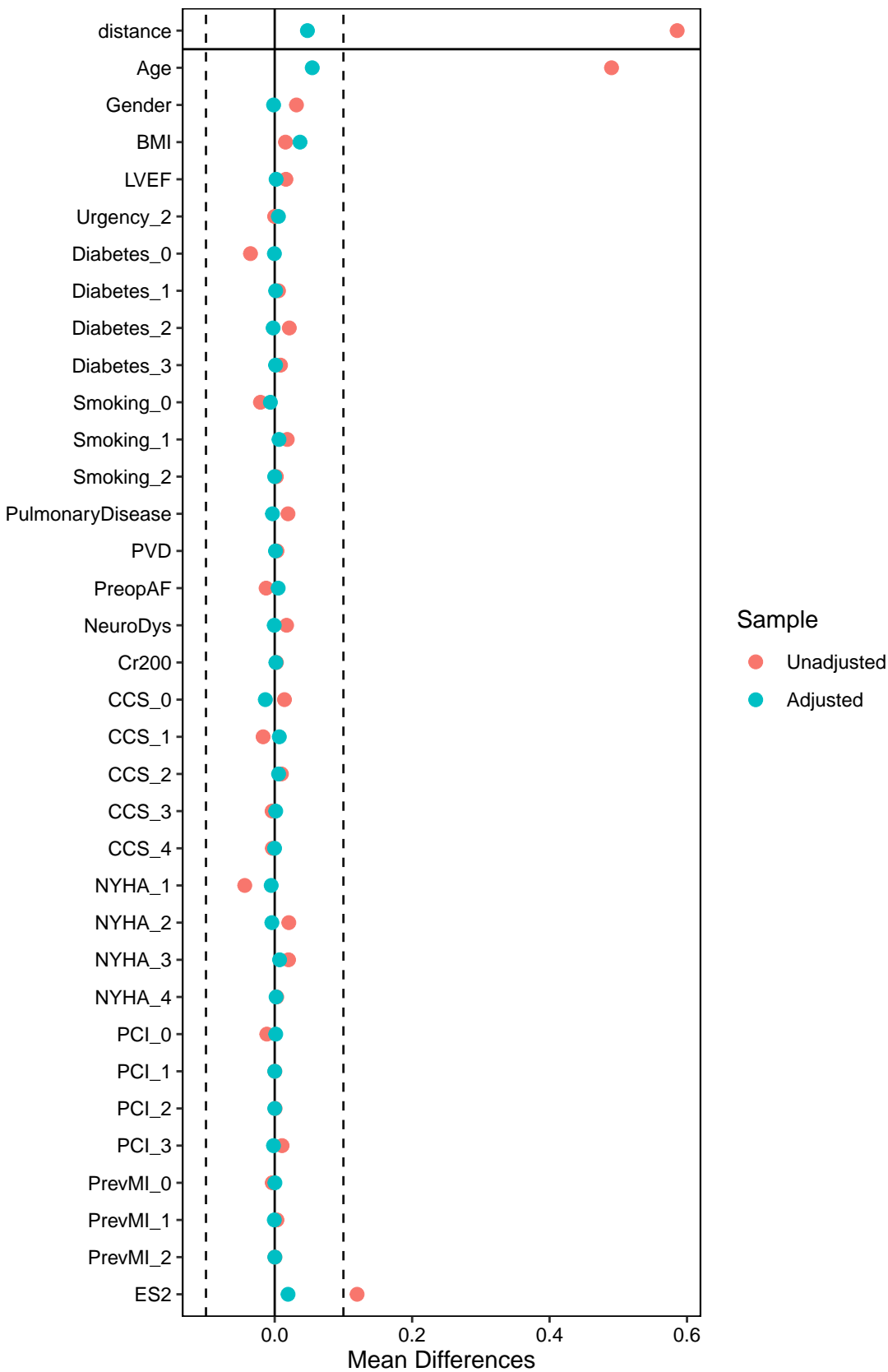

Supplement: Supplementary file 1 [file Image_1.pdf]
